# LONGITUDINAL TRANSITIONS IN FRAILTY STATES OVER SIX-YEAR FOLLOW-UP: RESULTS FROM THE KFACS

**DOI:** 10.1093/geroni/igae098.4020

**Published:** 2024-12-31

**Authors:** Heeeun Jung, Hyung Eun Shin, Daehyun Lee, Jae Young Jang, Hyunjin Cho, Nahyun Lim, Chang Won Won, Miji Kim

**Affiliations:** Kyung Hee University, Seoul, Republic of Korea; Kyung Hee University, Seoul, Republic of Korea; Kyung Hee University, Seoul, Republic of Korea; Kyung Hee University, Seoul, Republic of Korea; Kyung Hee University, Seoul, Republic of Korea; Kyung Hee University, Seoul, Republic of Korea; Kyung Hee University, Seoul, Republic of Korea; Kyung Hee University, Seoul, Republic of Korea

## Abstract

The longitudinal nature of frailty reveals its dynamic process through transitions of frailty states over time. However, the transitions between frailty states, including their progression and reversibility, remain unclear in Korea. We investigated these longitudinal transitions in frailty states among Korean community-dwelling older adults. We conducted a 6-year follow-up analysis of 2,633 older adults (52.0% women; mean age 76.5 ± 3.9 years) from the Korean Frailty and Aging Cohort Study (KFACS). Participants who had frailty status measured at baseline and experienced at least one frailty state transition during follow-up were included. Frailty states are categorized as follows: robust, pre-frail, and frail according to the Fried frailty phenotype and death. Multi-state Markov models estimated 1-year transition probabilities and explored associations between sociodemographic factors (age, sex, education level) and frailty transitions. Over a mean follow-up period of 4.6 years, a total of 5,942 frailty state transitions were observed. Of these, 3,568 (61.5%) were maintained, 1,358 (22.9%) worsened, and 926 (15.6%) improved. The 1-year progression probability to pre-frail was higher than the probability of moving to frail (20.2% vs. 0.9%) in the robust state. Pre-frail individuals were more likely to revert to robust than frail individuals (17.7% vs. 2.6%). Furthermore, the 1-year transition to death was highest for those in the frail state compared to other states. Frailty progression was associated with older age, being female, and lower education level. In conclusion, understanding the dynamic changes between frailty states and the sociodemographic factors influencing these changes may offer opportunities to reverse frailty progression.

